# Very high and low residual spenders in private health insurance markets: Germany, The Netherlands and the U.S. Marketplaces

**DOI:** 10.1007/s10198-020-01227-3

**Published:** 2020-08-29

**Authors:** Thomas G. McGuire, Sonja Schillo, Richard C. van Kleef

**Affiliations:** 1 Department of Health Care Policy, Harvard Medical School, Boston, USA; 2 Institute for Health Care Management and Research, CINCH, University of Duisburg-Essen, Duisburg, Germany; 3 Erasmus School of Health Policy & Management, Erasmus University Rotterdam, Rotterdam, The Netherlands

**Keywords:** Health insurance, Risk selection, Risk adjustment, Risk sharing, I11, I13

## Abstract

We study the extremely high and low residual spenders in individual health insurance markets in three countries. A high (low) residual spender is someone for whom the residual—spending less payment (from premiums and risk adjustment)—is high (low), indicating that the person is highly underpaid (overpaid). We begin with descriptive analysis of the top and bottom 1% and 0.1% of residuals building to address the question of the degree of persistence in membership at the extremes. Common findings emerge among the countries. First, the diseases found among those with the highest residual spending are also disproportionately found among those with the lowest residual spending. Second, those at the top of the residual spending distribution (where spending exceeds payments the most) account for a massively high share of the unexplained variance in the predictions from the risk adjustment model. Third, in terms of persistence, we find that membership in the extremes of the residual spending distribution is highly persistent, raising concerns about selection-related incentives targeting these individuals. As our results show, the one-in-a-thousand people (on both sides of the residual distribution) play an outsized role in creating adverse incentives associated with health plan payment systems. In response to the observed importance of the extremes of the residual spending distribution, we propose an innovative combination of risk-pooling and reinsurance targeting the predictively undercompensated group. In all three countries, this form of risk sharing substantially improves the overall fit of payments to spending. Perhaps surprisingly, by reducing the burden on diagnostic indicators to predict high payments, our proposed risk sharing policy reduces the gap between payments and spending not only for the most undercompensated individuals but also for the most overcompensated people.

## Introduction

Health care spending is non-negative and right skewed with the top 10% and even more so the top 1% of spenders accounting for a disproportionate share of all spending. The National Institute for Health Care Management (NICHM) found, for example, using data from the Medical Expenditure Panel Survey for 2014 that the top 5% of spenders accounted for half of all spending, and the top 1% alone accounted for more than 20% of all spending.^1^ Bakx et al. [1] uncovered a similar pattern in The Netherlands where the top 1% of spenders accounted for one-quarter of all spending. For private health insurance in Germany, Karlsson et al. [10] show that 53% of all medical spending is due to the top 10% of all spenders.

Research focus on the high spenders is motivated not only by concern about cost, but also by a concern for the efficient functioning of individual health insurance markets organized around principles of choice and competition. In these market-based policies, competing plans receive a risk-adjusted payment for each enrollee, as is done in Germany, The Netherlands, Switzerland, the Marketplaces in the U.S., and elsewhere.^2^ Risk-adjusted payments fall far short for some individuals with very high spending, and it is this shortfall, not the level of spending per se, that creates incentive problems in these markets. Recently, research has sharpened the focus to what is termed high “residual spending”, where residual spending is the shortfall, spending less payment.^3^ Focus on residual spending also directs attention to the opposite case, when payments exceed spending.^4^ Very high profits at the individual level as well as very high losses can disturb the efficient functioning of health insurance markets, especially when these profits and losses are persistent, pointing out the importance to understand the population on both sides of the residual spending distribution.

Following recent papers, we focus on the extremely high and low residual spenders, conducting analyses on not just the top and bottom 1% of residuals, but also on the top and bottom 0.1%. Our main interest is in the question of whether membership in the extremes persists year-to-year. If so, strong adverse selection incentives are created by the predictable losers and predictable winners in an insured population which may lead insurers to selectively target profitable people while underserving the unprofitable ones (typically those with high medical needs). As our results show, in all three countries, these one-in-a-thousand people (on both sides of the residual distribution) play an outsized role in creating adverse incentives associated with health plan payment systems.

To build up to the question of persistence, we conduct descriptive analyses of the extremes of the residual-spending distribution. In spite of significant differences in health care systems and the risk adjustment algorithms employed in the three countries, some common findings emerge. First, the diseases found among those with the highest residual spending are also disproportionately found among those with the lowest residual spending. In other words, some of the health conditions that put individuals in the highly undercompensated category are also responsible for putting them in the highly overcompensated category. For example, in the U.S. Marketplaces, diabetes is the single most common illness among the most undercompensated and the most overcompensated. Second, in all three countries, those at the top of the residual spending distribution (where spending exceeds payments the most) account for a massively high share of the unexplained variance in the predictions from the risk adjustment model. This finding indicates that some form of reinsurance can have a substantial impact on payment system performance.

Our focus on persistence of high and low residual spending is distinct from much of the prior literature which has focused on high spenders and persistence of high spending, not residuals. For example, Hirth et al. [8] use 2003–2008 MarketScan employer claims data and find that 43.4% of those in the top 10% of health care spending in 2003 were in the top decile 1 year later. Some persistence remains even after 5 years. Of those in the top 10% in 2003, 34.4% were in the top decile 5 years later. Other studies in the U.S. also find persistence in spending.^5^ Karlsson et al. [10] for Germany and Bakx et al. [1] for the Netherlands characterize persistence in spending in privately insured populations.^6^ Van Veen [22] is one of the few studies with a focus on *residual* spending. Using data from the Netherlands, she finds that people in the top of the residual spending distribution in the current year have a relatively high probability of being in that same position next year. With a focus on residual spending, however, it is not only the positive extreme (i.e., underpayments) of the distribution that is relevant, but also the negative extreme (i.e., overpayments). As we will show, overpayments are sometimes very large in absolute value. In sum, we go beyond existing research to conduct comparative analyses with recent data from three prominent social health insurance markets to characterize patterns of residual spending on both extremes of the distribution.

After establishing the empirical importance and persistence of extremely high and low residual spending, we study how risk sharing can help to better compensate insurers for people with extremely high and persistent positive residual spending. Building on prior research from all three countries,^7^ we propose a new targeted form of risk sharing: residual-based reinsurance for persons with high residual spending in a prior period, a policy that, in effect, combines elements of high-cost risk-pooling and reinsurance. Results for the three countries are very similar. Targeted reinsurance reduces underpayments for these high-risk groups while touching a small share of overall spending and a very small share of the population, alleviating potential concerns with loss of plan incentives to control costs. And notably, although our targeted reinsurance is directed to reducing underpayment for high-cost cases, in all three countries, targeting also reduces overpayments for those for whom payments exceed costs the most. With targeted reinsurance in place, payment weights on very expensive illnesses are reduced, lowering overpayments for those with these serious illnesses. Similarity of the results for the three countries lends support to the generalizability of our findings.

“Health plan payment in Germany, The Netherlands and the U.S. marketplaces” describes the health plan payment systems and the data from the three countries. “Data and empirical methods” describes the methods and “Results” presents the results for several empirical analyses, beginning with estimation of risk-adjusted payment models faithful to actual practice in each country. These payment models form the basis for our analyses of residual spending and the simulation of our targeted risk sharing policy. “Discussion” concludes with a discussion of the findings from our empirical work and the payment system simulations.

## Health plan payment in Germany, The Netherlands and the U.S. marketplaces

Individual health insurance markets in Germany, The Netherlands and Marketplaces in the U.S. are organized around principles of regulated (or managed) competition, as first proposed by Enthoven [5]. Regulated competition puts health plans in competition for enrollees with the goal of generating incentives for cost containment and efficient plan design.^8^ In policies that differ country-by-country, regulators promote competition by allowing health plans limited discretion about plan design (e.g., in terms of provider network and cost-sharing options). At the same time, the regulators use demand- and supply-side pricing policies to guarantee public objectives such as individual affordability and accessibility of health plans. In all three countries, enrollee premiums do not differ according to the health status of individuals while some form of risk adjustment of plan payment is done centrally to transfer funds to plans enrolling costlier individuals. Risk adjustment is designed to ensure plan viability, but more importantly, to counter plan incentives to selectively attract the healthy and deter the sick from joining the plan.

### Germany

The public health insurance system in Germany is the largest individual health insurance market in the world, both in terms of the number of lives covered and in terms of the total plan payments [16, 23]. In 1996, free choice of sickness funds was introduced for all members of the social health insurance system. Two years prior, in 1994, risk adjustment was established to provide equal opportunities for sickness funds with diverging risk profiles of their insured. In 2009, the formerly mostly demographic risk adjustment system became morbidity based. Since then the payments to the sickness funds are calculated by an individual-level least squares regression weighted by the fraction of the year the individual is enrolled in the social health insurance system. Risk adjustors (see Table 1) are included in the form of dummy variables. The model is prospective: expenditures from 1 year are explained by demographic characteristics from the same year but the morbidity characteristics are taken from the previous year.^9^ From 2002 until 2009, risk adjustment was complemented by reinsurance from a high-expenditure pool through which sickness funds were reimbursed 60% of spending above a certain threshold. With the introduction of the morbidity-based risk adjustment, the high-expenditure pool was abolished. Starting in 2021, a high-spending pool will be reintroduced into the German risk adjustment system, compensating for 80% of individual-level spending above a threshold of 100,000 Euros.

**Table 1:** Health Plan Payment in Germany, The Netherlands and the U.S. Marketplaces

	Germany (2018)	The Netherlands (2018)	Marketplaces (2019)
Number of people covered	72.2 m	17.1 m	10.6 m
Average plan spending per person per year	3034 €	2504 €	$5772 (silver plan benchmark average premium 2018)
Geographic market	National	National	State with sub-state rating areas
Number of plans	110	About 60 (varying by premium and contracted care; each plan can come with deductible options and group arrangements)	1–15, mean 4.2 varies by rating area
Premiums	Single premium per health plan	Single premium per plan; rebates for voluntary deductibles and group arrangements	Limited age bands
Risk adjustment data	Morbidity data from 2017; spending data from 2018. Interim payments are made prior to final reconciliation	Spending from 2015 (made representative for 2018, e.g., in terms of benefits package and projected spending)	2017 MarketScan data on large employers/insurers
Risk adjustment demographics	Age, sex, reduced earning capacity, reimbursement status	Age, sex, regional factors, socioeconomic factors, yes/no institutionalized, level of education	Age, sex, geography
Risk adjustment disease indicators	201 hierarchical morbidity groups (HMG) based on: Prescribed drugs and in- and outpatient diagnoses	124 morbidity indicators based on: Prescribed drugs (PCGs) Hospital diagnoses (DCGs) Physiotherapy diagnoses Mental care diagnoses Durable medical equipment Multiple-year high or low spending 1-year spending on home care	128 Hierarchical Condition Categories (HCCs) based on ICD-10 inpatient and outpatient diagnoses Plus 12 RXC groups based on drug claims Extensive interactions
Timing of risk adjustment disease indicators	Prospective (i.e., disease indicators are based on information from the prior year)	Prospective (i.e., disease indicators are based on information from one or multiple prior years)	Concurrent (i.e., disease indicators are based on data from the same year as spending predictions)
Risk adjustment estimation procedure	Weighted least squares	Weighted least squares	Weighted least squares
Risk adjustment comments	Separate model for sick leave payments	Separate models for somatic care, mental health care and out-of-pocket spending below the mandatory deductible	Separate models for age groups and tiers of coverage
Risk sharing	Reinsurance 2002–2008 60% above threshold	Reinsurance until 2014; risk corridors until 2016	Transfer formula embeds reinsurance of 60% above threshold of $2 m
*R*^2^ from the risk adjustment regression	26%	32% for somatic care 23% for mental healthcare 33% for OOP spending	35%

Due to the volume of information presented here notes for each element are not provided. There are some additional features of the payment systems in each country not contained in the table, for example, Germany has special rules for those living abroad and for a small number of individuals paid by cost reimbursement. For detailed descriptions of each of these payment models with much of the information covered here, see Wasem et al. [23], Van Kleef et al. [21] and Layton et al. [12]

### The Netherlands

Since 2006, The Netherlands have had a national health insurance system based on principles of regulated competition. Consumers may switch insurance plans every year and insurers have several tools to promote efficiency such as selective contracting of healthcare providers, utilization management and flexibility regarding provider payment design [21]. The Dutch risk adjustment system has been improved over time. In the early years, the risk adjustment system was supplemented with reinsurance to mitigate selection incentives remaining after risk adjustment and to mitigate plans’ business risk due to financial uncertainties surrounding specific healthcare system reforms. As risk adjustment was improved and the health insurance market stabilized, reinsurance thresholds were increased; in 2014, reinsurance was abolished altogether.^10^ For our analyses we use the Dutch risk adjustment system from 2018, which consisted of three different models, one for each of the following categories: somatic care, mental health care, and out-of-pocket payments due to the mandatory deductible of 385 Euros per adult per year [21]. For simplicity, our analyses will be based on the model for somatic care only. This model accounts for about 85% of total spending and includes a broad set of risk adjustors based on several types of information, which are described in Table 1. Risk adjustors take the form of dummy variables indicating whether an individual is a member of a class or not. Risk adjustor coefficients for the model of 2018 were derived by an individual-level weighted least squares regression of annualized expenditures in 2015 on demographic variables from year 2015 and the disease indicators listed in Table 1 from 2014 or before. Like Germany, the Dutch model is, therefore, also prospective, using morbidity data from a prior period to predict spending in the current period. Prior to estimation of the risk adjustment model 2018, some modifications were applied to make the available data from 2015 representative for 2018 (e.g., including modifications for changes in the benefits package).^11^

### U.S. Marketplaces

The U.S. Marketplaces, created as part of the Affordable Care Act (2010) and popularly known as “Obamacare”, began enrolling individuals and families in 2014 [14, 15]. These markets, organized at the state level, are intended to provide affordable health insurance for those without insurance through their employers or through other public programs. The law included a number of reforms which shifted the individual health insurance market toward a version of regulated competition, including income-related subsidies, (partial) community rating of premiums, mandated coverage of a basket of “essential health benefits,” and guaranteed issue and renewal provisions prohibiting plans from rejecting applicants based on their health status. As of 2019, about 11.4 million Americans are enrolled in a Marketplace plan, the majority of whom receive some premium subsidy. The extent of coverage in Marketplace plans ranges from approximately 60% on average for “bronze” plans to 90% for “platinum” plans. The most popular metal level is “silver” with coverage at 70%.

The Marketplace risk adjustment model assigns risk scores to enrollees based on their demographics and observed diagnoses during the current plan year (i.e., calendar year), in contrast to the programs in Germany and The Netherlands which use morbidity data from the previous year. The Marketplace model is said to be “concurrent” as opposed to “prospective” in the other two countries. Risk scores are calculated using a model developed by the Department of Health and Human Services (HHS), the HHS Hierarchical Condition Categories (HHS-HCC) model. See Table 1. The HHS-HCC model has undergone several iterations since its inception in 2014, with HHS-HCC V0519 (2019), a slight modification of V0518 (2018), introduced for 2019.^12^ The HHS-HCC V0519 model predicts an enrollee’s medical spending by mapping diagnoses coded on insurance claims into one of 128 HHS-selected HCCs, which were drawn from the larger set of HCCs available in the diagnostic classification system.^13^ In a major change, V0518 added 12 drug categories (RXC01–RXC12) of which ten (RXC01–RXC10) are used directly in the risk adjustment model; with the other two used for HCC and RXC interactions only. The V0519 drops the RXC11–12 interactions. Drug variables are generated using National Drug Codes (NDC) from pharmacy claims with prescription filled dates within the benefit year (NDC from medical claims are not accepted).^14^ Beginning with V0418 (2017), CMS introduced a variable measuring “months of enrollment” during a contract year to contend with possible underpayment for those with partial enrollment periods.^15^

A “temporary” reinsurance component was part of the Marketplace payment system in the first 3 years, but due to a continuing concern about high-cost cases, a modest reinsurance function was restored through changes in the formula transferring funds among health plans (Jost [9, 13]).^16^ In this paper, we estimate weights using the V05-2019 HHS-HCC model for risk adjustment.^17^

## Data and empirical methods

Our empirical methods consist of a series of steps. First, we estimate the current risk-adjusted health plan payment model in each country, following as closely as possible actual estimation practices, and use this to calculate residual spending for each individual in the data. Data from Germany used in this paper are from one large insurer.^18^ For each individual, information on diagnoses and expenditures from all hospital visits and outpatient treatments are available. Expenditure data are available for filled prescriptions at the person level. Data from The Netherlands are those actually used for calibration of the risk adjustment model of 2018 and includes individual-level information on medical spending and risk characteristics for the entire population under the Dutch basic health insurance of 2015 (*N* = 17 m). This information comes from various administrative sources, including insurers, the tax collector and the registration service for social benefits. The U.S. data are a more recent version of the MarketScan data used to calibrate plan payment models in the Marketplaces. Our 6.8 million sample from MarketScan uses the same exclusion/inclusion criteria as used by HHS in estimating risk adjustment models, as has been done in previous research on Marketplace payment models.^19^ We estimate a model for adults only, with total spending the dependent variable. Months of enrollment is not included since, contrary to the Dutch and German data, we restrict our sample to those enrolled for the full year. Table 2 summarizes some information about the data in all three countries. Many more people have some morbidity indicator in Germany, 51.6%, as compared to the other two countries. In the U.S. Marketplaces, the 22.3% figure means that almost 80% of the population has no diagnosis used for payment during a year. These no-indicator people are paid on the basis of age and gender alone. In all countries, the distribution of spending is highly skewed, with a maximum observed spending in 1 year at € 2.8 m and € 1.8 m in Germany and The Netherlands, respectively, and $8.5 m in the U.S. Marketplaces. We regard it as particularly notable that some of our findings presented in the Results section are common across the countries in spite of the differences in payment models used (described in Table 1) and the underlying population and spending characteristics (described in Table 2).

**Table 2:** Data from three countries

	Germany	The Netherlands (somatic care only)	U.S. Marketplaces
Source	Sample of a nationwide operating sickness fund	Insurers and government agencies	Large employers/insurers
Number of individuals	2.4 million	17.0 million	6.8 million
Year	2016	2015	2017
Age range	Entire population	Entire population	21–64
Average age	49.4	41.3	44.9
Female proportion	N/A	50.6%	52.2%
Proportion with any HCC/morbidity flag	51.6%	27.0%	22.3%
Average number of HCCs/morbidity flags	1.5	0.5	0.3
Spending distribution			
1st percentile spending	€ 0	€ 3	$0
10th percentile spending	€ 115	€ 90	$0
90th percentile spending	€ 7900	€ 4602	$14,085
99th percentile spending	€ 38,560	€ 32,241	$80,974
Maximum Spending	€ 2,859,088	€ 1,834,548	$8,541,629

U.S. data only cover people with full-year enrollment. Data from Germany and The Netherlands also cover people who were enrolled only part of the year; percentiles of spending presented here are based on actual spending (rather than annualized spending). The positive spending at the 1st percentile in The Netherlands is a mandatory fee everyone pays to register with a practitioner. People with partial-year enrollment pay this mandatory fee in proportion to the fraction of the year they were enrolled. For Germany, the insurer supplying the data requested we not do report the proportion of female in the population

In a second step, we conduct parallel descriptive analyses to characterize people in the very top/bottom of the residual-spending distribution for all three countries. More specifically, we identify and analyze the following groups: bottom-0.1%, bottom-1%, top-1%, and top-0.1%. Our analyses focus on patterns in healthcare spending and disease indicators. These descriptive analyses provide a first taste of the extent to which extremely low/high residual spenders differ from the rest of the population. Moreover, these analyses check to what extent patterns of spending and disease flags in these groups are similar across countries.

In a third step, we track residual spending year-to-year in each country to examine the extent to which ‘being a low/high residual spender’ is predictable and/or persistent, features that contribute to selection incentives. For both top and bottom groups, we calculate (1) the probability of an individual to reoccur in the same group next year and (2) the correlation between residual spending this year and the next. In addition, we calculate mean residual spending (i.e., under/overcompensation) this year for deciles of residual spending last year.

Given the finding that membership to the top and bottom groups is highly persistent, we explore how a targeted form of reinsurance can help to mitigate selection incentives regarding these groups (step four). Whereas traditional reinsurance compensates insurers for a share of individual-level spending above a certain threshold of spending, our form of reinsurance targets payments to those with high residual spending rather than high spending per se. Residual-based reinsurance has been proposed and applied by Schillo et al. [19]. In this paper, having identified those with predictable high residual spending as the main source of concern, we take targeting reinsurance one step further, directing reinsurance to those with high probability of high residual spending, i.e., those who had very high residual spending in the previous year. Residual-based reinsurance with eligibility based on very high residual spending from the previous year renders this new policy a combination of “high risk-pooling” as proposed by Van Barneveld et al. [20] and “residual-based reinsurance” as first proposed by Schillo et al. [19]. We simulate the effects of this new policy on selection incentives using the following metrics: group-level under/overcompensation, “Payment System Fit” (PSF) and “Cumming’s Prediction Measure” (CPM). PSF is an *R*^2^-type statistic (analogous to a pseudo-*R*^2^) that recognizes that the payment a plan receives for an individual, $R_{i}$, can include other components in addition to the predicted spending from a risk adjustment model. It quantifies the proportion of squared residual spending from a payment system relative to that of a system that provides insurers with a flat payment per enrollee equal to the mean per person spending in the population. In the case where payments do not include components outside the regular regression, PSF equals *R*^2^.^20^ Due to its squaring property, PSF (like the *R*^2^) is sensitive to outliers. CPM does the same but then for absolute residual spending and is thus less sensitive to outliers. Our linear CPM also incorporates payments via risk sharing as well as predictions from the regression model.

Like any form of risk sharing, our targeted form of reinsurance is expected to reduce incentives for cost control since it links (residual) spending and health plan payments: for those in the targeted group whose residual spending exceeds a threshold, health plan payments go up with (residual) spending. Incentives for cost control with the non-linear risk sharing features of both conventional and residual-based reinsurance are not readily described with a single number. We track funds required and people touched in our simulation results to shed light on how our risk sharing policy affects cost-control incentives. To avoid overfitting issues regarding our measures of payment fit and incentives for cost control, we follow a split-sample approach. For each country, we use one half of the sample, chosen at random, to estimate the risk adjustment and reinsurance parameters and the other half to calculate our outcome measures.

## Results

This section presents the results of our analyses and is structured as follows. We first display the findings from our descriptive analysis regarding spending patterns and disease indicators in the top and bottom groups (“Characterizing extremely low/high residual spenders”). After that, we continue with our findings regarding the persistence of residual spending (“Persistence”) and the effects of our new targeted form of reinsurance (“Targeted reinsurance for dealing with predictably high and low residual spending”).

### Characterizing extremely low/high residual spenders

#### Risk adjustment and residual spending

Table 3 presents summary statistics from the regressions as well as the distribution of residual spending—spending less predicted value—computed after the risk adjustment estimation. Our *R*^2^ estimates for Germany, 23.1%, The Netherlands, 32.1% and the U.S. Marketplaces, 36.8%, are similar to those in other reports from each country, 24.6% for Germany [4], 32.1% for the Netherlands [21], and 41% for the U.S. Marketplaces.^21^ A higher *R*^2^ for the Marketplace model compared to that for Germany or The Netherlands is expected because Marketplaces use a concurrent risk adjustment model rather than the prospective models used in the other two countries.

**Table 3:** Regression results from three countries

	Germany	The Netherlands (somatic care only)	U.S. Marketplaces
*R*^2^	0.231	0.321	0.368
CPM	0.244	0.319	0.312
Distribution of residuals	(€)	(€)	($)
Minimum	− 364,094	− 466,752	− 545,865
Percentile 0.1	− 27,842	− 24,539	− 95,335
Percentile 1.0	− 11,442	− 8881	− 29,848
Percentile 50	− 802	− 436	− 1582
Percentile 70	− 147	− 254	− 504
Percentile 80	129	377	1121
Percentile 99	27,009	20,282	50,652
Percentile 99.9	87,494	70,636	189,918
Maximum	2,512,588	1,815,707	11,260,784

In all three countries, risk adjustment leaves some individuals highly underpaid and others highly overpaid. Table 3 also shows the spending values associated with selected percentiles of the residual spending distribution. Negative residual spending (spending less revenues) corresponds to overpayment, with the greatest negative values of − € 364 k Euros in Germany, − € 467 k in The Netherlands, and − $546 k in the U.S. The minimum and maximum values of a distribution are determined by a single observation, so it is more telling to compare the values at the top and bottom 1% and 0.1% of the distributions. On both sides of the distribution of residual spending, the U.S. is characterized by higher absolute values, while the German and Dutch results are broadly similar. The 0.1% of the distribution occurs at − € 28 k and − € 25 k for Germany and The Netherlands, respectively, and the much larger − $95 k for the U.S. Marketplaces. The results imply, for example, that 0.1% of the Dutch population are overpaid by more than € 25 k.

On the other side of the residual distribution, there is again a rough equivalence between the German and Dutch results with the U.S. Marketplaces being more extreme. Specifically, the German and Dutch 99.9% values are € 87 k and € 71 k, respectively, whereas the U.S. is $190 k. The top and bottom 1% can also be seen in Table 3. One percent of the population in the U.S. Marketplaces are underpaid by the concurrent system by $51 k or more. Spending remains less than revenues until around the 80th percentile of the distribution in all countries, another indication of the skewness in the distribution of residual spending. Risk adjustment reduces, but does not eliminate, the skewness in health care spending.

#### Health conditions in the extremes of the residual spending distribution

For each country, Table 4 shows the five most prevalent disease indicators among the one in a thousand most undercompensated people. In Germany (Panel A), the flag for diabetes appears in 14.4% of these extremely high residual spenders. The table also shows the frequency of the indicator in the entire population and the rank and prevalence among those who are the most overcompensated. In Germany, the disease with the highest prevalence among the most undercompensated (hypertension) is ranked second among the most overcompensated. For all five disease indicators, the prevalence in both tails of the residual distribution is vastly greater than the prevalence in the entire population.

**Table 4:** Disease indicators among the most (0.1%) undercompensated in three countries

Indicator	0.1% under-compensated		0.1% over-compensated		Total population		
	Rank	Prev. (%)	Rank	Prev. (%)	Rank	Prev. (%)	Share of unexplained variance (%)
Panel A: Germany (top 5 out of 192 disease indicators)							
HMG091 (hypertension)	1	19.4	2	20.4	1	15.7	17.5
HMG019 (diabetes)	2	14.4	5	16.5	2	7.1	12.7
HMG071 (polyneuropathy)	3	12.1	4	16.9	9	3.1	10.5
HMG080 (heart failure)	4	11.8	3	17.1	6	4.0	12.1
HMG058 (severe depression)	5	10.6	8	13.5	3	6.5	9.6
Panel B: The Netherlands (top 5 out of 76 disease indicators)							
PCG7 (high cholesterol)	1	11.6	13	9.9	1	6.1	11.5
PCG14 (hearth disease)	2	10.8	2	22.9	8	2.1	11.6
sDCG1 (cluster of about 30 diseases)	3	9.6	21	6.7	2	3.1	9.3
sDCG3 (cluster of about 20 diseases)	4	8.4	4	18.4	24	0.6	7.6
DCG7 (cluster of about 20 diseases)	5	7.8	5	17.6	28	0.5	6.9
Panel C: U.S. Marketplaces (top 5 out of 94 disease indicators)							
G01 (diabetes)	1	22.2	1	33.7	1	7.5	22.8
HCC008 (metastatic cancer)	2	15.3	5	23.4	22	0.2	10.7
HCC130 (congestive heart failure)	3	13.8	6	21.3	8	0.8	13.0
G15 (COPD)	4	12.9	7	17.0	2	4.7	11.9
G13 (respiratory arrest)	5	12.8	4	25.4	21	0.3	13.7

The last column of Table 4 reports the “share of unexplained variance” associated with people with this disease indicator. In Germany, those with the indicator for polyneuropathy (3.1% of the population) account for 10.5% of the unexplained variance associated with the risk adjustment model. In other words, this relatively small portion of the population, even in the presence of a disease indicator for this condition, is responsible for a relatively large share of the unexplained variance after risk adjustment. To scale this variance differently, if this portion of the variance was explained instead of unexplained, it would increase the *R*^2^ of the risk equalization model to 31.2%.^22^ Each of the top five illnesses among the most undercompensated is associated with a large share of the unexplained variance, a result common across our three countries.^23^

In The Netherlands, the most common disease indicator among the top 0.1% of residual spenders is the PCG for ‘high cholesterol’. For this indicator, and even more so for the other Dutch indicators in Table 4, the prevalence among the most undercompensated is (much) higher than that in the total population. Apparently, despite their above-average predicted spending, people flagged by these indicators have a relatively high probability of being extremely underpaid. Three of the most prevalent indicators among the highest residual spenders are also present in the top-5 indicators among the lowest residual spenders. This is remarkable since payment weights (not shown here) for these indicators are not among the highest in the risk adjustment model. It must be true that some people in these groups are also flagged by other disease indicators (with high payment weights). In line with their high prevalence at both ends of the residual spending distribution, all five indicators presented here make a substantial contribution to the variance in spending not explained by the Dutch risk adjustment model.

The most common disease indicator among the top residual spenders in the U.S. is the group code for diabetes, seen among 22.2% of the very most undercompensated. Diabetes is also the most prevalent code among the most overcompensated; indeed, one in three of the bottom residual spenders has this flag. The commonality of illnesses on both tails of the residual distribution is indicated by the rankings (1, 5, 6, 7, 4) of the most prevalent among the most undercompensated appearing in the most overcompensated. Again, as in Germany and The Netherlands, those with these illnesses are responsible for large shares of the unexplained variances.

For most disease indicators in Table 4, the prevalence among the most overcompensated is greater than that among the most undercompensated. An explanation for this is that to be extremely overcompensated, people need to be flagged by one or more (very expensive) disease indicators, which is not true for the other side of the residual spending distribution. As a result, disease flags are expected to be more present among people with low residual spending than among those with high residual spending.

#### Share of spending on drugs

**Fig. 1:**
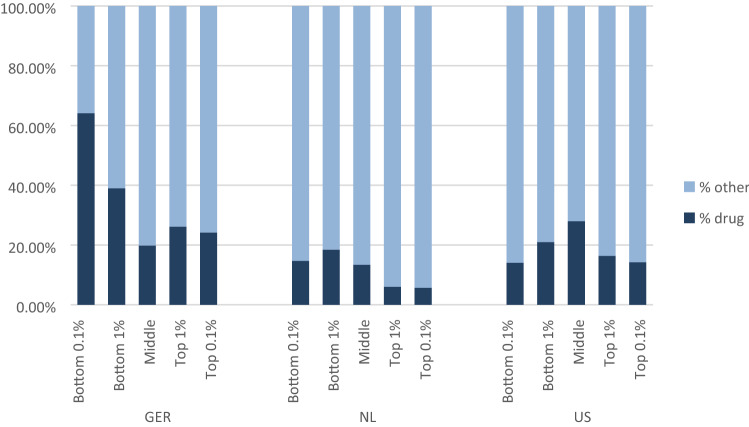
Share of spending on drugs by residual spending groups

In addition to the patterns in disease flags among low and high residual spenders, we are also interested in how types of spending vary across the distribution of residual spending and across countries. Because of differences in the way utilization is classified in the datasets available for this study (for example, whether hospital outpatient claims are classified as “hospital” as in The Netherlands or “Outpatient” as in the U.S.), we focus here on the share of spending on drugs outside the hospital reported similarly in all countries. Figure 1 shows the share of spending on drugs in all spending by position in the residual spending distribution (the bottom and top 0.1% groups are included in the bottom and top 1% groups). Here the patterns differ somewhat across the countries. Germany has the highest share of spending on drugs with the bottom 1% group spending nearly 40% on drugs. The bottom 0.1% group has an even higher share of spending on drugs: it reaches 64%. The Netherlands shows the lowest share of spending on drugs. The top 1% group only spends 6% on drugs—the top 0.1% group spends about the same. For the U.S. Marketplaces, the bottom 0.1% and the top 0.1% group each spend about 14% on drugs. The bottom and top 1% groups are similar as well, spending 21% and 16%, respectively, on drugs. In the U.S., it is the middle group that has the highest share of spending on drugs at 28%.

Figure 1 may convey the appearance that spending on drugs in the U.S. is less than in Germany, but the opposite is true. The middle group is by far the biggest in each country, and the figure reports percentages rather than absolute amounts. The average spending on drugs outside the hospital in the U.S. across the entire population is $1717, whereas it is €770 in Germany. The Dutch spend the least overall, at €271 per capita. In the U.S. and Germany, drug spending is based on prices paid at retail outlets, and does not take into account manufacturer rebates, which will be important for branded drugs, particularly in the U.S. In the Netherlands drug spending is corrected for rebates.

#### Unexplained variance

**Fig. 2:**
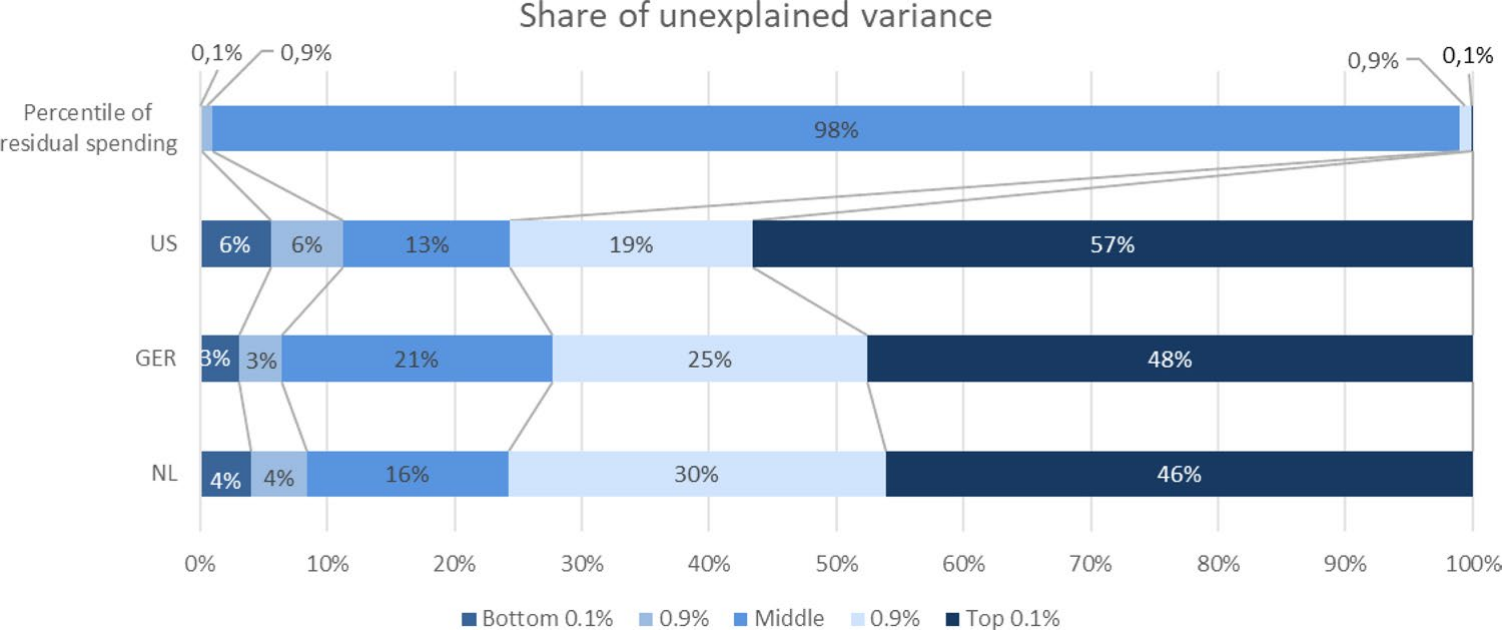
Share of unexplained variance per percentile of residual spending

Figure 2 shows where the variation in residual spending falls along the distribution of residual spending. The results are remarkably similar across countries.^24^ Consider first Germany, and start with the top 0.1% of residual spenders, the very most underpaid group. While the share of spending for this group is about 5%, the share of unexplained variance in spending is 47.5%. In other words, almost half of the residual sum of squares after risk adjustment for the entire population rests with this one-in-one-thousand group.^25^ Considering the top 1.0% (in which the top 0.1% is included) brings us to 18.5% of total spending and 72.3% of variance unexplained by the risk adjustment model. The issue of “fit” of Germany’s risk adjustment model as measured by unexplained variance is seen to be largely an issue of fit in the extreme upper tail of the residual spending distribution. The situations in The Netherlands and the U.S. Marketplaces are very much the same. The top 0.1% of the residual spending distribution accounts for about half of the unexplained variance, whereas the top 1.0% accounts for three quarters.

### Persistence

If being grossly under- or overpaid occurred at random, under- and overpayment would affect financial uncertainty for health insurers but would not create selection-related incentives, since a plan would have no action that it might take that would be correlated with high profits or losses. From the standpoint of selection incentives, the degree of persistence in membership in the tails of the residual spending distribution is important to quantify. If people tend to stay in these very unprofitable or very profitable groups, plans will have a powerful incentive to deter the former and attract the latter.

Table 5 measures persistence in two ways. Again, start with Germany and the top 0.1% group in terms of residual spending. If membership in this group were random, only 0.1% of people in this group would reappear in the top 0.1% of residual spending next year. Instead, 20.7% remain in the same top 0.1% group year-on-year, a likelihood 207 times greater than would be expected by pure chance. Results for this group for the U.S. are much the same, a simple persistence of 27.2% year-on-year retained membership. The Dutch are different, with “only” 10.6% remaining year-to-year, which is likely due to the Dutch risk adjustors defined on the basis of “spending persistence”. Still, more than 10% of the 0.1% top Dutch residual spenders returning to the group for a second year imply very significant persistence in residual spending. Persistence in group membership on both sides of the residual spending distribution is evident for all three countries. We include the large middle group for reference, but it is not surprising that most people in the wide band of what we call “middle” remain in that band year-to-year.

**Table 5:** Measures of Persistence in Residual Spending in Three Countries

	Germany	The Netherlands (somatic care only)	U.S. Marketplaces
Share of people reoccuring in same group next year			
Bottom 0.1%	27.1%	10.6%	30.3%
Bottom 1.0%	30.5%	17.7%	35.3%
Middle	98.8%	97.2%	98.6%
Top 1.0%	17.5%	13.0%	23.9%
Top 0.1%	20.7%	10.6%	27.2%
Correlation between residual spending in this year and the next			
Bottom 0.1%	0.30	0.32	0.14
Bottom 1.0%	0.33	0.19	0.17
Middle	0.14	0.05	0.21
Top 1.0%	0.21	0.29	0.54
Top 0.1%	0.26	0.33	0.58

We also measure persistence by simple correlation of costs from one year to the next, with the groups set by membership in a top or bottom tail group in the initial year. If there was complete regression to the mean, the year-to-year correlation would be zero, but in fact we see reasonably high correlations of around 0.3 for both Germany and The Netherlands. The U.S. Marketplaces exhibit a slightly different pattern with a lower correlation for the groups most overpaid and a higher correlation of around 0.6 for the groups underpaid.

**Fig. 3:**
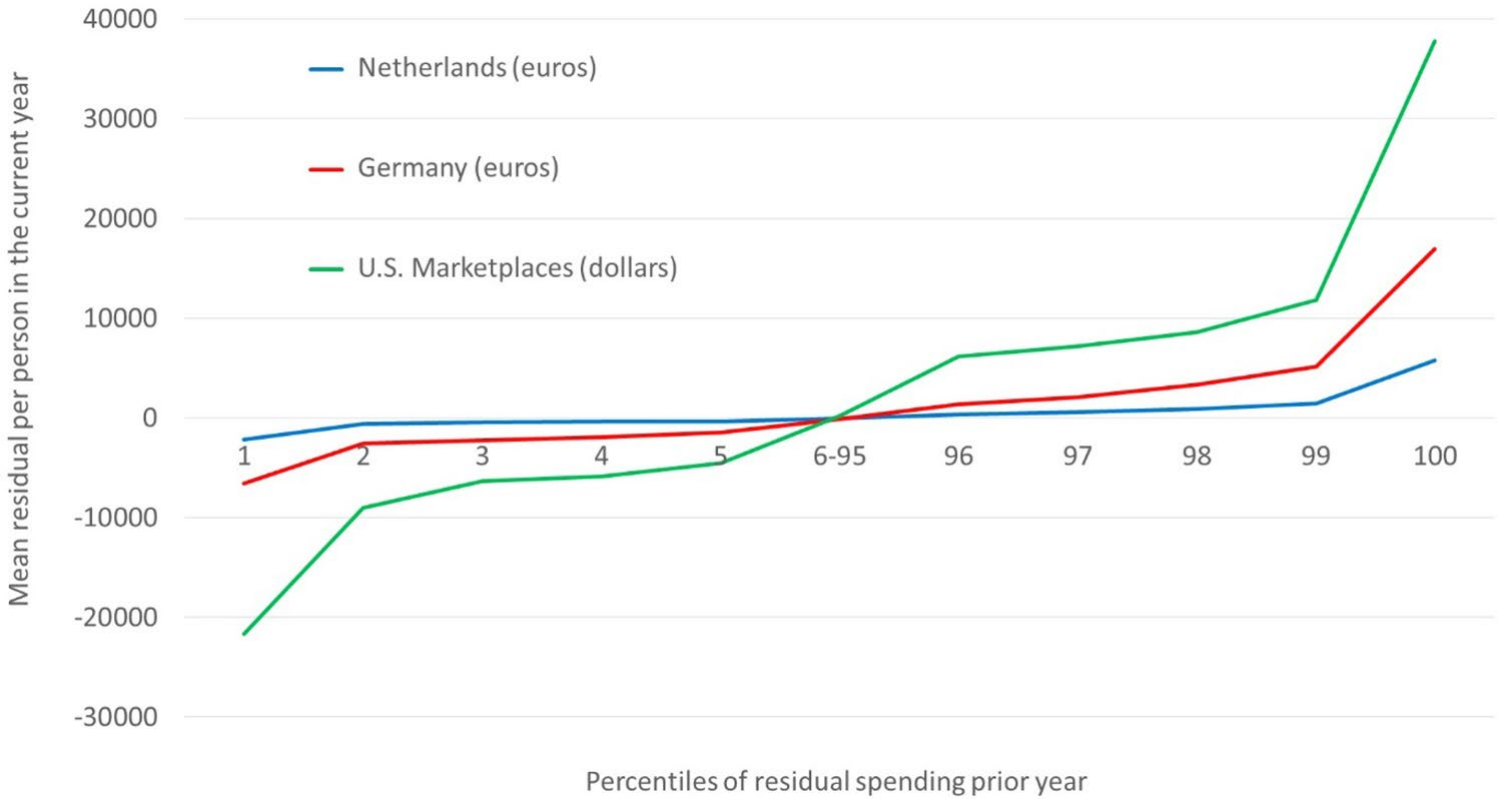
Mean residual spending for groups defined by residual spending in year *t* − 1: three countries

Figure 3 presents persistence from another angle: mean residual spending this year for groups defined by residual spending in the prior year. At the far left are those in the lowest percentile of residual spending in the prior year, i.e., the most overpaid. The next group consists of those in the second-lowest percentile, and so on. The figure presents five groups corresponding to the bottom percentiles (left) and five groups corresponding to the top percentiles (right). The middle group (i.e., 6–95) contains all people who were between the 6th and 95th percentile of the residual spending distribution in the prior year. The data series show that the extremely low residual spenders in the prior year are on average profitable to insurers in the current year. The opposite holds for extremely high residual spenders in the prior year; these people tend to be (very) unprofitable in the current year. The variation in profitability among the presented groups is highest for the U.S. and lowest for The Netherlands.

### Targeted reinsurance for dealing with predictably high and low residual spending

The motivation and working of our targeted reinsurance policy can be explained by reference to Fig. 3. In all three countries (the absolute value of) mean residual spending in the current year is largest for the group on the far right, i.e., those who were in the top-1% of the residual spending distribution in the prior year. This is the group we target with residual-based reinsurance. Specifically, our proposed form of risk sharing pays reinsurance based on residual spending in this group with a sufficiently low threshold to cap the mean residual spending for our targeted group to the average level in the neighboring group, i.e., those between the 98th and 99th percentiles of the residual-spending distribution in the prior year.

To improve the overall fit of payments to spending, we optimize risk adjustment weights for the presence of risk sharing and vice versa. In other words, our payment weights are chosen to best fit payments to spending given the presence of our targeted reinsurance, and our residual-based reinsurance uses residuals from the optimized weights. An iterative procedure is needed because a change in risk adjustment payments affects the mean underpayment in both our group of interest (i.e., the one to the very right of Fig. 3) and the neighboring group, calling for a modification of the reinsurance threshold to level the mean underpayment for these two groups. For all three countries, ten iterations are sufficient to converge on a joint solution for the optimal weights and residual spending threshold.

**Fig. 4:**
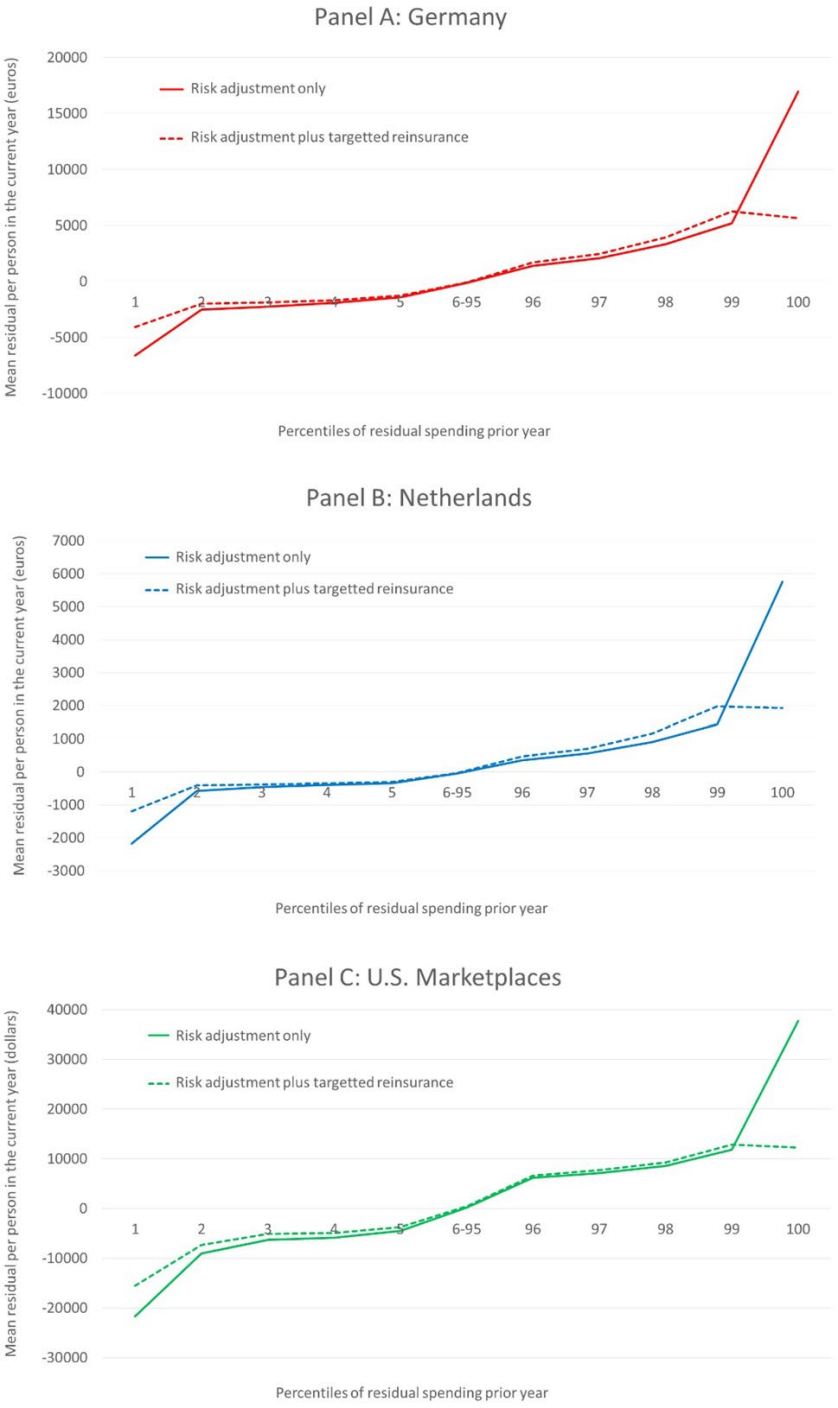
Mean residual spending for groups defined by residual spending in year *t* − 1: three countries/two models

The three panels in Fig. 4 show the effects of our targeted reinsurance system on the outcomes for the groups defined by residual spending in the prior year. These groups mimic those presented in Fig. 3 for each country. In each panel, the solid line corresponds to the relevant country line in Fig. 3. Note, however, that the scale of the vertical axis is now different for the three countries. As intended, the reinsurance system caps the mean undercompensation of people in the highest percentile of residual spending in the prior year to that of those in the second-highest percentile.^26^ Perhaps surprisingly, reinsurance targeted at the extreme right of the residual spending distribution substantially reduces overpayments at the extreme left of the distribution. The explanation, previewed in Table 4 above, is that the disease indicators most prevalent among the most undercompensated tend also to be prevalent among the most overcompensated. Intuitively, risk sharing directed to the undercompensated reduces the burden on the diseases of the undercompensated to fit the higher health care costs, resulting in lower estimated payment weights for these diseases. It was the high payment weights on these diseases that created the extremely overcompensated. Reducing the payment weights, thus, improves the situation on the left extreme as well as the right extreme side of the residual spending distribution. Additional payments to those most undercompensated must come from somewhere, and, in effect, optimizing the risk adjustment weights means that financing of payments for the undercompensated comes from just where you would want it to come from—the overcompensated.

In sum, the payment system simulations show that our targeted form of reinsurance mitigates both predictably low and predictably high residual spending. In addition to the group-level outcomes presented in Fig. 4, we also calculated two measures of individual-level fit, i.e., PSF and CPM. The outcomes are presented in Table 6 and show that targeted reinsurance comes with a (substantial) increase in individual-level payment fit. In all three countries, the increase in PSF is larger than that in CPM, the explanation being that our targeted form of reinsurance inherently allocates payments to those people for whom payment gaps from risk adjustment are largest.^27^ In the U.S. the increase in individual-level fit is larger than in Germany and The Netherlands, which can be explained by the fact that the distribution of residuals in the U.S. is even more skewed than in the other two countries. The share of unexplained variance (Fig. 2) for the top-0.1% in the U.S. is 57%; whereas in Germany and The Netherlands, it is 48%, respectively, 46%. This also means that the reinsurance funds (needed to cap the mean underpayment in our group of interest) is somewhat larger in the U.S. Marketplaces than in the other two countries (as we will see next).

**Table 6:** Outcomes for two Alternative Payment Systems

	Germany	The Netherlands (somatic care only)	U.S. Marketplaces
Risk Adjustment (RA) only			
Mean residual year *t* for:			
Top-1% residual spenders in *t* − 1	€16,960	€5,764	$37,761
Bottom-1% residual spenders in *t* − 1	− €6606	− €2172	− $21,656
PSF	0.232	0.319	0.379
CPM	0.246	0.318	0.312
RA + targetted reinsurance			
Mean residual year *t* for:			
Top-1% residual spenders in *t* − 1	€5664	€1923	$12,284
Bottom-1% residual spenders in *t* − 1	− €4084	− €1197	− $15,521
PSF	0.479	0.436	0.626
CPM	0.291	0.338	0.360
Reinsurance threshold	€21,062	€27,063	$40,538
Share of spending affected by reinsurance	0.036	0.019	0.043
Share of population affected	0.003	0.001	0.003

To shed light on how our reinsurance policy affects incentives for cost control, Table 6 also presents the share of funds required for our reinsurance policy and the share of people touched by this policy. For the Netherlands, we find that insurers receive a reinsurance payment for 0.1% of the population (one in a thousand); the share of reinsurance payments in total spending equals 1.9%. For Germany, these figures equal 0.3% and 3.6% and for the U.S. Marketplaces they are 0.3% and 4.3%. In all three countries, the share of payments necessary to fund our targeted reinsurance is small. The number of people affected is very small, ranging from 0.1 to 0.3% of the population.

## Discussion

The three countries studied here all rely on managed competition for all or part of their social health insurance system, and all use a sophisticated disease-based risk adjustment algorithm to pay insurers. Indeed, the risk adjustment schemes in these three countries are arguably the most complex and sophisticated algorithms in use anywhere. Nonetheless, the payment formulas differ in important ways. The Marketplace formula is concurrent rather than prospective as in Germany and The Netherlands. The number and form of morbidity-based indicators varies considerably. The health care systems differ too, in the populations included, depth of coverage, forms and extent of managed care, costs of various inputs, patterns of health care, and so on. For example, the share of spending on drugs is much greater in the U.S. than in The Netherlands. In spite of these many profound differences, and remarkably in our view, our three-country comparisons identify several important findings that hold in all settings.

In all three countries, risk adjustment leaves some individuals highly underpaid and others highly overpaid. In Germany and The Netherlands, one in a thousand people are underpaid by more than € 87 k and € 71 k, respectively. With a residual of > $190 k for this top-0.1% group, underpayments in the U.S. Marketplaces are even more extreme. On the other side of the residual distribution, we find that one in a thousand people are overpaid by at least € 28 k (Germany), € 25 k (The Netherlands) and $95 k (U.S. Marketplaces). In all three countries, the top- and bottom-1% groups share some of the same diseases. With risk adjustor weights estimated with least squares, as is done in all three countries, the sum of residuals conditional on a disease indicator is zero. People with a disease indicator who tend to be very underpaid, thus, must be balanced with people with the same disease indicator who are overpaid. Although it is not necessarily true that the balancing overpayment comes from people with extreme overpayment (i.e., instead it could come from many people with less-extreme overpayment), diseases disproportionally found among the most undercompensated tend to be also disproportionally found among the most overcompensated.

Another finding common in all three countries is that the one in a thousand highest residual spenders are responsible for a large share of the variance in residual spending, from 46.1% in The Netherlands to 47.5% in Germany and 56.6% in the U.S. Marketplaces. In other words, almost half of the residual sum of squares after risk adjustment for the entire population rests with the top 0.1% of most underpaid people. If this portion of the variance was explained instead of unexplained, it would increase the *R*^2^ of the risk adjustment models to more than 60%. This finding is behind the huge impact of reinsurance policies on squared measures of individual-level payment fit.

When it comes to the effects of extreme residual spending on the functioning of health plan markets, our most relevant finding is that being grossly under- or overpaid does not occur at random. For all three countries, we find that extreme under- and overpayments are persistent. For people in the top 1% of losses this year, insurers can expect a mean underpayment next year of €16,960 (Germany), €5764 (The Netherlands) and $37,761 (U.S. Marketplaces). For the one in a hundred most overpaid people this year, insurers can expect a mean *over*payment next year of €6606 (Germany), €2172 (The Netherlands) and $21,656 (U.S. Marketplaces). These findings indicate that extreme under/overpayment is to some extent predictable and can contribute to selection problems.

The high degree of persistence in membership in the extremes of the residual spending distribution in all three countries raises concerns that insurers might take steps to deter those who tend to be underpaid and attract those who tend to be overpaid. Attracting the healthy/deterring the sick among subsets of the populations with the disease indicators (such as diabetes) prevalent on both extremes of the residual spending distribution could be a highly profitable strategy, and potentially lead to distortions in the efficient care for these groups. In response to these findings, we proposed a form of reinsurance, based on residuals, and targeted to members of a “risk pool” defined on past-year very high undercompensation. Careful targeting (along with re-estimating the beta weights in risk adjustment to take into account the reinsurance payments) leads to very substantial improvements in overall fit of payments to spending, with especially large effects for the most extremely under- and overcompensated. The share of people affected by this form of risk sharing is very small, less than 3 in 1000 in all three countries. While our proposed policy seems effective in better tying payments to spending, there are alternative approaches to the same issue. One example would be to find ways to split groups like those with diabetes and other illnesses prevalent among the undercompensated into those likely to be on one or the other side of the residual spending distribution. Calling attention to the powerful effects members of the tails of the residual distribution have on the overall fit of the models is the first step in directing policy attention to these important groups.

Cross-country data analyses are a powerful way to compare effects of health plan payment systems on incentives for insurers, and, in particular, to seek results that are likely to be generalizable to other data and policy settings. Our study shows, however, that this type of research comes with challenges related to the underlying differences in the health care systems. Differences go deeper than simple differences in risk equalization models, down to coding conventions and treatment practices. In some ways, analyses for Germany and The Netherlands are more comparable to one another than they are to the U.S. Marketplaces. The healthcare systems themselves are quite similar in the two European countries. The payment system in the Marketplaces is concurrent rather than prospective. And unlike in Germany and The Netherlands where data from actual experience are used for figuring risk equalization payments, in the U.S., data for calibrating the risk equalization model are from large employers and insurers, not from the Marketplaces themselves. Recognizing these important differences makes the commonality of our findings even more striking.

## Footnotes

1 https://www.nihcm.org/categories/concentration-of-us-health-care-spending.

2 Belgium, Colombia, Israel, and Medicare Advantage (the private option for Medicare beneficiaries in the U.S.) among other countries and sectors, share some similar features. McGuire and Van Kleef [16] contain descriptions of the individual health plan markets structured as regulated competition in 14 countries and sectors. In the three markets studied here, payments to insurers consist of two components: a compensation from the risk adjustment system and a premium. For reasons of simplicity, however, this paper integrates the two components by assuming that the payment that an insurer receives for individual *i* equals *i*’s predicted spending from the risk adjustment model. For all three markets, this payment will closely approximate the sum of *i*’s premium and compensation from the risk-adjusted system.

3 Schillo et al. [19], Farid and McGuire [6], Kauer et al. [11], Van Veen [22]. “Residuals” have, in fact, been the focus of empirical risk adjustment research all along. An *R*^2^ of a risk adjustment regression is based on residuals, as are measures of over- and undercompensation and predictive ratios at a group level. An over/undercompensation measure is, in a system in which payment is fully determined by risk adjustment, simply the average value of the residuals for the group in question.

4 In any break-even payment system, residual payments must sum to zero. Furthermore, risk-adjusted payments based on an OLS/WLS regression with disease indicators implies that residuals sum to zero conditional on a disease indicator. This property of OLS/WLS-based payment models guides some of our interpretation below.

5 Monheit [18] found that the top 1% of spenders account for 27% of all expenditure, and between 1996 and 1997, of the top 5% of spenders 30% stay within that group in the next year. Similarly, Figueroa et al. [7] use a 20% sample of Medicare beneficiaries from 2012 to 2014 and find that 28.1% of individuals are in the top 10% of spending for three consecutive years. Using data from the Medical Expenditure Panel Study (MEPS) Cohen and Yu [2] observe 40% of those in the top decile of spending in 2009 remain in the top decile in 2010. For Medicaid/Chip insured people, persistence was shown to be even higher: DeLia [3] calculated that of the top 1% of spenders in 2011, 31% remained there 3 years later and 27% were among the top 1% spenders in each of the years from 2012 to 2014. If deceased and disenrolled persons were excluded from the analyses, the corresponding percentage would be 47.3%.

6 Karlsson et al. [10] find that 56.19% of the insured in the top quintile of spending in 2010 are also in the top spending quintile in 2011. Over the whole study period from 2005 to 2011, 55.02% remain within the top spending quintile. Bakx et al. [1] were able to show for the entire Dutch population that 60% (56%) of individuals in the top quintile of the spending distribution in 1 year are also in the top quintile of spending after 1 (2) year.

7 Van Barneveld et al. [20], Schillo et al. [19] and McGuire et al. [17].

8 By ‘health plan competition’, we mean competition among health insurers who offer one or multiple health plans. A ‘health plan’ refers to a health insurance product. All consumers who have the same ‘health plan’ have an identical contract with the same insurer concerning benefits coverage, cost-sharing, quality, services, etc. Since objectives and strategies of insurers can differ across health plans (primarily in the U.S. and The Netherlands), this paper will speak of health plans instead of insurers as decision makers.

9 The German regression is run on cost per day which is in principle equivalent to the annualization procedure used in the Netherlands.

10 As of 2020, some reinsurance will be reintroduced for mental healthcare spending.

11 In the regression model, expenditures were annualized and the observations weighted by the fraction of the year an individual was enrolled in 2015 (which can be smaller than 1.0 due to birth, death, migration and other factors). For example, a person with a half-year enrollment and 2000 Euro expenditures was given a weight of 0.5 and annualized expenditures of 4000 Euro (2000/0.5).

12 https://www.cms.gov/CCIIO/Resources/Regulations-and-Guidance/Downloads/Updated-CY2018-DIY-instructions.pdf. V0518 split HCC037 (chronic hepatitis) into a 37a and 37b to distinguish types of hepatitis and, in a major change, included risk adjustor variables based on prescription drug data.

13 Some of these 128 HCCs are further grouped in the regression model. They are also used to form interaction terms. Eight HCCs are categorized as “severe illnesses” and if a patient has any of these eight severe illnesses, they receive a SEVERE flag. This SEVERE variable interacts with 16 other HCCs or groups of HCCs to create 16 interactions, nine of which belong to the high-cost category and the other seven to the medium-cost category. The patient gets an additional flag added to their risk score for having any of the high-cost interactions or medium-cost interactions. If they have both then only the high-cost flag is added. In total, there are 94 morbidity-related variables used in V0519. Both V0518 and V0519 make extensive use of interactions among the HCC variables. For an overview of the HCC variables and interaction terms, see https://www.cms.gov/CCIIO/Resources/Regulations-and-Guidance/Downloads/2019-Updtd-Final-HHS-RA-Model-Coefficients.pdf.

14 When an NDC from a pharmacy claim is not available, HCPCS codes (Healthcare Common Procedure Coding System) from inpatient, outpatient, and professional medical claims with discharge dates or through dates within the benefit year can be used to create drug indicators. All our observations include drug coverage so we use only NDC codes to create drug variables.

15 https://www.cms.gov/CCIIO/Resources/Forms-Reports-and-Other-Resources/Downloads/RA-March-31-White-Paper-032416.pdf. See pages 35–39.

16 As of August, 2018, seven states in the U.S. have received waivers from the federal government to reintroduce additional reinsurance features in their Marketplaces. https://www.commonwealthfund.org/blog/2018/affordable-care-act-under-trump-administration?omnicid=EALERT1465357&mid.

17 Software to implement V05-2019 was recently released and can be found at https://www.cms.gov/CCIIO/Resources/Regulations-and-Guidance/index.html. HHS estimates risk adjustment weights without regard to the fact that reinsurance affects plan obligations, implying that the regression weights are not optimal for predicting plan spending obligations net of reinsurance payments. The present reinsurance is set at such a high threshold that any difference in estimated weights would be trivial. In “Targeted reinsurance for dealing with predictably high and low residual spending” where we make more use of the reinsurance function, we optimize regression weights for the presence of reinsurance.

18 More description of the data source is contained in Schillo et al. [19].

19 See Layton et al. [12], and Layton and McGuire [13]. Following practice for estimating risk adjustment models in the Marketplaces, our sample is restricted to those individuals who had both prescription drug and mental health coverage and who had no negative or capitated claims.

20 In that case we will use *R*^2^ throughout this paper.

21 CMS reports the *R*^2^ of V0519 estimated on MarketScan data for 2014 and 2015 to be 41%. https://www.cms.gov/CCIIO/Resources/Regulations-and-Guidance/Downloads/2019-Updtd-Final-HHS-RA-Model-Coefficients.pdf. In spite of some differences in implementation of V0519, the correlation between our predictions and those using CMS coefficients is a reassuring 0.96.

22 Given the current *R*^2^ of 0.231 (see Table 3), a reduction of 10.5% in the unexplained variance would imply an increase in *R*^2^ to 0.231 + 0.105 × (1 − 0.231) = 0.312.

23 Membership in the groups is not mutually exclusive, so the percentages of unexplained variance cannot simply be “added up”.

24 In results not shown, the share of spending across the range of residual spending is also similar in the three countries. The share of spending accounted for by the top 0.1% and 1.0% of the residual distribution ranges from 5 to 7% and 19 to 24%, respectively, in the three countries.

25 *R*^2^ is the share of total variance explained by the risk adjustment model, and is normally taken as a useful statistic indicating good performance of a risk adjustment system. The share of unexplained variance for a risk adjustment model can also be readily calculated. From Table 3 we know that the risk adjustment model for Germany has an *R*^2^ of 23.1%, leaving 76.9% “unexplained.” The top 0.1% accounts for 47.5% of the unexplained variance, or 35.9% of the total variance in spending. For an earlier paper that recognized the massive share of the unexplained variance in the Marketplaces, see Farid and McGuire [6]. Similar findings for Switzerland have also been reported. See Kauer et al. [11].

26 A closer look at Fig. 4 reveals that the mean undercompensation of people in the highest percentile of residual spending is slightly lower than that of those in the second-highest percentile. The explanation for this is that our risk adjustment and reinsurance parameters are based on the training sample (in which the mean undercompensation in the two groups after reinsurance is exactly the same), while the results in Fig. 4 are based on our test sample (where the undercompensation in the two groups is subject to random variation). It appears that some regression to the mean in spending is somewhat higher in the very tail of the distribution, accounting for the slightly lower undercompensation in all three countries for this group.

27 Due to its squaring property, PSF is more sensitive to (reductions in) large payment gaps than CPM.
